# Evaluation of the current guidelines for antibacterial therapy strategies in patients with cirrhosis or liver failure

**DOI:** 10.1186/s12879-021-07018-2

**Published:** 2022-01-04

**Authors:** Yuzhu Dong, Dan Sun, Yan Wang, Qian Du, Ying Zhang, Ruiying Han, Mengmeng Teng, Tao Zhang, Lei Shi, Gezhi Zheng, Yalin Dong, Taotao Wang

**Affiliations:** 1 Department of Pharmacy, The First Affiliated Hospital of Xi’an Jiaotong University, Xi’an, 710061 China; 2 Department of Pharmacy, The Third Affiliated Hospital of Chongqing Medical University, Chongqing, 401120 China; 3 Department of Pharmacy, The Second Affiliated Hospital of Xi’an Jiaotong University, Xi’an, 710061 China; 4 Department of Infections, The First Affiliated Hospital of Xi’an Jiaotong University, Xi’an, 710061 China

**Keywords:** Cirrhosis, Liver failure, Clinical practice guideline, Bacterial infections, AGREE II

## Abstract

**Background:**

Bacterial infections are common complications in patients with cirrhosis or liver failure and are correlated with high mortality. Clinical practice guideline (CPG) is a reference used to help clinicians make decisions. This systematic appraisal aimed to evaluate the methodological quality and summarize the recommendations of reported CPGs in these patients.

**Methods:**

We systematically searched CPGs published from 2008 to 2019. The methodological quality of the included CPGs was assessed using the AGREE II instrument. We extracted and compared recommendations for prophylactic and empirical treatment strategies.

**Results:**

Fourteen CPGs with a median overall score of 56.3% were included. The highest domain score was Clarity of Presentation (domain 4, 85.4%), and the lowest was for Stakeholder Involvement (domain 2, 31.3%). Three CPGs had an overall score above 80%, and 6 CPGs had a score above 90% in domain 4. Prophylaxis should be strictly limited to patients with varicose bleeding, low ascites protein levels and a history of spontaneous bacterial peritonitis. Fluoroquinolones (norfloxacin and ciprofloxacin), third-generation cephalosporins (G3) (ceftriaxone and cefotaxime) and trimethoprim–sulfamethoxazole (SXT) are recommended for preventing infections in patients with cirrhosis or liver failure. G3, β-lactam/β-lactamase inhibitor combinations (BLBLIs) and carbapenems are recommended as the first choice in empirical treatment according to local epidemiology of bacterial resistance.

**Conclusions:**

The methodological quality of CPGs focused on patients with cirrhosis or liver failure evaluated by the AGREE II instrument is generally poor. Three CPGs that were considered applicable without modification and 6 CPGs that scored above 90% in domain 4 should also be paid more attention to by healthcare practitioners. Regarding recommendations, norfloxacin, ciprofloxacin, ceftriaxone, cefotaxime, and SXT are recommended for prophylactic treatment appropriately. G3, BLBLIs, and carbapenems are recommended for use in empirical treatment strategies.

**Supplementary Information:**

The online version contains supplementary material available at 10.1186/s12879-021-07018-2.

## Introduction

Bacterial infections are very common complications in patients with cirrhosis or liver failure, with a 30-day mortality rate ranging from 30 to 50% [1, 2]. In the management of bacterial infections, patients with gastrointestinal bleeding and ascites are at high risk of infections, and prophylactic use of antibiotics is necessary. For example, cirrhotic patients with ascites are prone to several complications including spontaneous bacterial peritonitis (SBP), which is the most frequent, life-threatening bacterial infection in these patients [3]. Additionally, delayed or inappropriate empirical antibiotic therapy correlates with higher mortality and the risk of emerging multidrug-resistant organisms resistant organisms (MDROs) [4, 5]. Furthermore, the presence of MDROs could lead to the failure of infections prophylaxis and empirical treatment [6]. This vicious cycle makes antibacterial strategies more complicated. Given the high mortality and drug resistance associated with bacterial infections, it is more urgent than ever to develop appropriate antibacterial strategies. However, the indications for prophylaxis and treatment schedules recommended by clinical practice guidelines (CPGs) are conflicting and confusing.

A CPG is an important reference used to help clinicians make clinical decisions. It has been reported that cirrhotic patients who receive a treatment adherent to CPG recommendations could benefit from good therapeutic efficacy [7]. Moreover, a multicenter evaluation study found that adherence to Baveno CPGs could improve the clinical outcomes of patients with acute variceal haemorrhage [8]. Several CPGs have been developed for the management of bacterial infections in patients with cirrhosis or liver failure. However, there is substantial heterogeneity in these CPGs and also in the scientific literature. Antibiotic strategies for prophylactic and empirical treatment vary among countries and regions according to the available CPGs for these patients, which has implications for the management of bacterial infections in clinical practice. To date, there has been no critique of the similarities, differences and contentious issues across these CPGs.

This systematic appraisal aimed to (1) evaluate the methodological quality analysis using a systematic critical appraisal approach and (2) summarize recommendations of current CPGs to identify the indication for prophylactic treatment and recommend antibiotics for prophylactic and empirical treatment in patients with cirrhosis or liver failure.

## Methods

### Search strategy

A systematic review of the Cochrane, PubMed, Embase databases, National Knowledge Infrastructure (CNKI) and five online guideline repositories [the National Institute for Health and Care Excellence (NICE), Guidelines International Network (GIN), Scottish Intercollegiate Guidelines Network (SIGN), National Health and Medical Research Council (NHMRC), and New Zealand Guidelines Group (NZGG)] was conducted to identify guidelines using Preferred Reporting Items for Systematic Reviews and Meta-Analyses (PRISMA) criteria. The search was confined to studies published within the last 11 years (2008–2019). The search terms and database search strategy are summarized in Additional file 1: Appendix S1. We also searched in Clinical Practice Guideline website of MedLive (http://www.medlive.cn/) manually with the term ‘liver cirrhosis or liver failure or hepatitis’ in Chinese to increase the spectrum of results.

### Study selection

All search records were exported to the EndNote X7 library, and duplicates were removed. We screened article titles/abstracts and the full text of relevant CPGs and included CPGs according to the following criteria: (1) CPGs must have been developed by a panel of multidisciplinary experts; (2) CPGs must be intended for application to adult patients; and (3) CPGs must include explicit recommendations for treating bacterial infections (prophylactic/empirical treatment) in patients with liver cirrhosis or liver failure. We excluded (1) CPGs for the management of hepatitis A/B/C; (2) CPGs for children/pregnant women/liver transplant patients/cystic fibrosis patients, and (3) CPGs focused on fungal infections.

### Data selection

All documents related to the CPGs (full CPG document, Additional file 1: Appendix S1 and Additional file 2: Appendix S2) were collected for analysis. We extracted and summarized the characteristics of the CPGs, including country of origin, year of publication, guideline developer and recommendations on antibacterial therapy. Three authors (Yuzhu Dong, Dan Sun, and Yan Wang) independently extracted data related to the Appraisal of Guidelines for Research and Evaluation (AGREE) II tool and discrepancies were resolved through discussion (Yuzhu Dong, Dan Sun, Yan Wang and Taotao Wang).

### Methodological quality appraisal of CPG development

Three reviewers (Yuzhu Dong, Dan Sun, and Yan Wang) evaluated each CPG independently using the AGREE II instrument (version December 2017). This evaluation tool consists of 23 items grouped within six domains [Scope and Purpose (domain 1), Stakeholder Involvement (domain 2), Rigor of Development (domain 3), Clarity of Presentation (domain 4), Applicability (domain 5), and Editorial Independence (domain 6)]. Each item is ranked on a seven-point scale (1 represents strongly disagree with, and 7 represents strongly agree with). The final item scores were combined to provide a scaled domain score (as a percentage). The domain score was calculated with the formula described in the AGREE II user’s manual guidance. Detailed information is available on the AGREE website (www.agreetrust.org).

### Assessment of CPG recommendations

One author (Yuzhu Dong) scrutinized each CPG and then summarized the key points and recommendations identified. The completeness and accuracy of the recommendation details were checked by another author (Dan Sun). Then, two other authors (Yan Wang and Qian Du) checked the CPG recommendations to ensure that they were classified into two treatment strategies, Namely “prophylaxis for bacterial infections” and “empirical treatment for SBP and bacterial infections other than SBP”. We selected the following 4 aspects to compare the content of the recommendations and to find similarities and differences across these recommended strategies: (1) indication for prophylactic treatment; (2) recommended antibiotics for prophylactic and empirical treatment; (3) principles of empirical treatment; and (4) information on dosage, frequency and duration.

### Statistical analysis

All data were analysed using the SPSS V.18.0 software. Median and interquartile range (IQR) for the domain scores and overall scores were calculated. We used the intraclass correlation coefficient (ICC) with a two-way mixed effects model to calculate the interrater agreement. ICCs were computed for each domain score and overall score. The level of agreement was classified as poor (ICC < 0.40), fair (ICC: 0.40–0.59), good (ICC: 0.60–0.74) or excellent (ICC: 0.75–1.00) according to a previous study [9].

## Results

### Characteristics of included CPGs

**Fig. 1:**
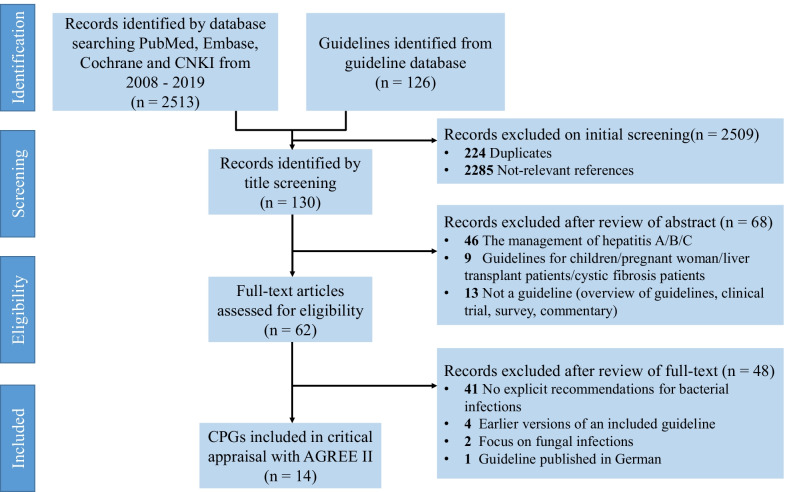
Flow diagram of studies identification and selection

The searches identified 2639 relevant records, and 14 CPGs [10–23] fulfilled the selection criteria and were scrutinized eventually (Fig. 1). The CPGs characteristics and their development methods are summarized in Table 1. Six CPGs were originally from Asia, 7 CPGs came from Europe, and only one was from USA. Most CPGs (n = 11) were developed by medical associations, research groups (n = 2), or expert panels (n = 3). The target groups of all 14 CPGs included physicians, while pharmacists and nurses were included in the multidisciplinary teams in only three CPGs. The methods to develop evidence mentioned in 9 of the 14 CPGs [10, 13, 15, 17–19, 21–23] were based on literature analysis. Meanwhile, two CPGs (AASLD 2012 and EASL 2010) combined the literature analysis with expert panel approaches to develop evidence and provide both data-supported and experience-based recommendations. The criteria used to grade evidence were heterogeneous, and the grading systems used for the appraised CPGs are shown in Additional file 2: Appendix S2. Grading of Recommendations Assessment, Development and Evaluation (GRADE) (n = 10) was main system adopted by all 14 CPGs.

**Table 1:** CPGs characteristics and development methods for recommendations

Guideline, year	Region/country	Developers	Target group	Journal	Multidiscipli-nary team	Grading system	Evidence based	Guideline review
EASL 2018 [10]	Europe	EASL	Physicians	J Hepatol	Hepatologists	GRADE	Systematic literature review	Peer review
CMA 2018 [11]	China	LFALG, CSID; CMA; SLDALG, CSH, CMA	Physicians	J Clin Hepatol	Hepatologists	GRADE	NR	NR
EASL 2017 [12]	Europe	EASL	NR	J Hepatol	Hepatologists	GRADE	NR	Reviewers
KASL 2017 [13]	Korea	KASL	Physicians	Dig Liver Dis	KASL Committee	GRADE	Systematic literature review	External review board composed of seven KASL members
CMA 2017 [14]	China	CSH, CMA	Physicians	Chin J Gastrointest Endosc	Hepatologists	GRADE	NR	NR
NICE 2016 [15]	The United Kingdom	NICE	Physician	–	Hepatologist, Nurse Specialist, Patient/Carer Member, Pharmacist Hepatology	GRADE	Systematic literature review	NR
CMA 2016 [16]	China	CSH, CMA; CSG, CMA; CSE, CMA	Physician	J Clin Hepatobiliary Dis	Hepatologists	GRADE	NR	NR
JSG 2015 [17]	Japan	JSG	NR	J Gastroenterol	Hepatologists	GRADE	Systematic literature review	Evaluation Committee
BSG 2015 [18]	The United Kingdom	Clinical Services and Standards Committee of BSG	Clinicians and healthcare professionals	Gut	Hepatologist, gastroenterologist, Member of BSG liver section, interventional radiologist; patient representative, research nurse; Nursing representative	Oxford	Systematic literature review	External peer reviewer
APASL consensus 2014 [19]	Asia	APASL	NR	Hepatol Int	Hepatologists	Oxford	Systematic literature review, expert panel	NR
APCCMID 2013 [20]	Asia-Pacific	The member of APCCMID	Clinicians	Hepatobiliary Pancreat Dis Int	NR	GRADE	NR	NR
AASLD 2012 [21]	America	AASLD	Physicians	–	AASLD Practice Guidelines Committee	ACC/AHA	Systematic literature review	NR
EASL 2010 [22]	Europe	EASL	NR	J Hepatol	Hepatologists	GRADE	Systematic literature review, expert opinion	Three reviewers
SIGN 2008 [23]	Scotland	SIGN	Acute physicians, gastroenterologists, gastrointestinal surgeons, endoscopists, pharmacists, anaesthetists and nurses	–	Gastroenterologist, Radiologist, Principal Pharmacist, Gastroenterology Nurse Practitioner, Haematologist, Physician	SIGN methodology	Systematic literature review	Specialist review

*CPGs* clinical practice guidelines, *EASL* European Association for the Study of the Liver, *CMA* Chinese Medical Association, *LFALG* Liver Failure and Artificial Liver Group, *CSID* Chinese Society of Infectious Diseases, *SLDALG* Severe Liver Disease and Artificial Liver Group, *CSH* Chinese Society of Hepatology, *KASL* The Korean Association for the Study of the Liver, *NICE* National Institute for Health and Care Excellence, *CSG* Chinese Society of Gastroenterology, *CSE* Chinese Society of Endoscopy, *JSG* Japanese Society of Gastroenterology, *BSG* the British Society of Gastroenterology, *APASL* Asian Pacific Association for the Study of the Liver, *APCCMID* Asia-Pacifc Congress of Clinical Microbiology and Infection Consensus, *AASLD* The American Association for the Study of Liver Diseases, *SIGN* Scottish Intercollegiate Guidelines Network, *GRADE*:Grading of Recommendations Assessment, Development and Evaluation, *ACC/AHA* the American College of Cardiology and the American Heart Association Practice Guidelines, *NR* not report

### Appraisal of the CPGs

**Fig. 2:**
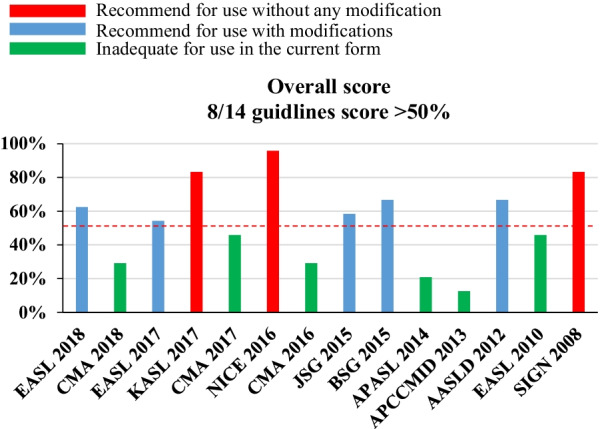
Overall score of guidelines with AGREE II. AGREE II: Appraisal of Guidelines for Research and Evaluation II

**Fig. 3:**
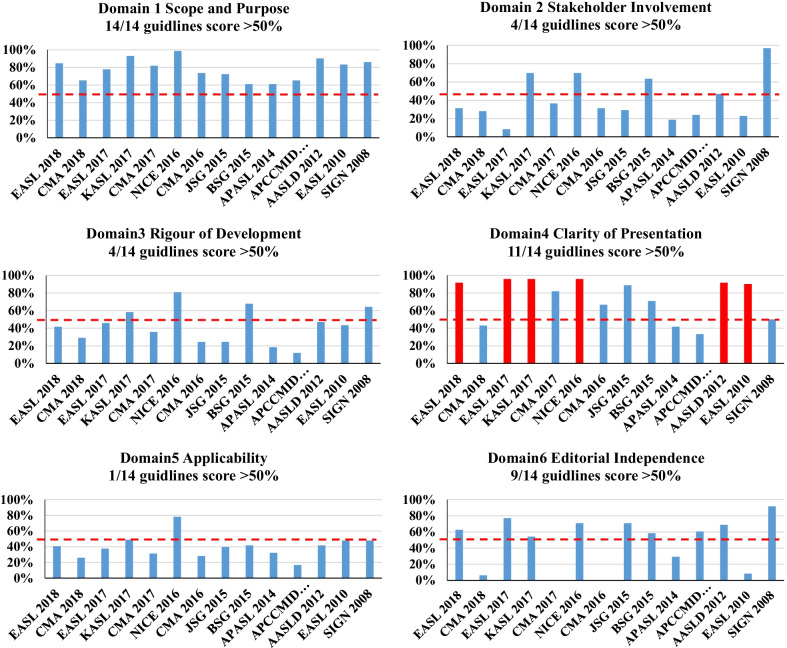
Domains of guidelines appraisal with AGREE II. AGREE II: Appraisal of Guidelines for Research and Evaluation II. Red color: clinical practice guidelines scored above 90% in the domain 4

The standardized median domain scores and overall score of AGREE II for 14 CPGs are summarized in Table 2 and Fig. 2. The median overall score for the 14 CPGs was 56.3% (IQR, 29.2–70.8%). The domains with the highest score were Clarity of Presentation (domain 4) (85.4%, IQR, 48.3–92.7%) and Score and Purpose (domain 1) (79.9%, IQR, 65.3–87.2%). The lowest domain score was Stakeholder Involvement (domain 2), with a median score of 31.3% (IQR, 23.7–65.1%). The scores of the other three domains (Applicability, Rigour of Development and Editorial Independence) ranged from 40.1% (IQR, 30.5–47.9%) to 59.4% (IQR, 7.8–70.8%). Interrater reliability was classified as excellent for all domains and overall scores (Table 2). Standardized domain scores for each domain across the 14 CPGs are shown in Fig. 3. As shown in Figs. 2 and 3, 3 of 14 CPGs [13, 15, 23] had an overall score above 80%. Five CPGs [10, 12, 17, 18, 21] had scores ranging from 50 to 80%. Three CPGs [11, 14, 16] from China, two CPGs [19, 20] from Asia-Pacific and one [22] from Europe scored less than 50%. When we focused on the antibacterial recommendations in the CPGs, we noticed that 6 CPGs [10, 12, 13, 15, 21, 22] scored above 90% in Clarity of Presentation (domain 4).

**Table 2:** Total scoring and inter-rater reliability for AGREE II domain and overall scores

Domain	Score [median (IQR)] (%)	ICC [median (95% CI)]
Scope and purpose	79.9 (65.3–87.2)	0.892 (0.755–0.961)
Stakeholder involvement	31.3 (23.7–65.1)	0.989 (0.975–0.996)
Rigour of development	42.6 (24.4–59.8)	0.984 (0.965–0.994)
Clarity of presentation	85.4 (48.3–92.7)	0.973 (0.939–0.990)
Applicability	40.1 (30.5–47.9)	0.936 (0.857–0.977)
Editorial independence	59.4 (7.8–70.8)	0.975 (0.942–0.991)
Overall score	56.3 (29.2–70.8)	0.969 (0.931–0.989)

*AGREE* The Appraisal of Guidelines for Research and Evaluation, *IQR* interquartile range, *ICC* intraclass correlation coefficients, *CI* confidence interval

### Prophylaxis recommendations

The prophylaxis recommendations from 11 CPGs [10, 11, 13, 14, 16–18, 20–23] are summarized in Table 3. Six indications for prophylactic treatment were mentioned in the reviewed CPGs. Seven CPGs [13, 16–18, 21–23] recommended antibacterial prophylaxis in cirrhotic patients with varicose bleeding, five CPGs [10, 13, 17, 21, 22] recommended it in patients with low ascites protein levels (or/and patients with severe hepatic dysfunction/renal insufficiency/hyponatraemia), and six CPGs [10, 13, 14, 17, 21, 22] recommended it in patients with a history of SBP. Two additional CPGs recommended prophylaxis in patients with chronic liver failure [20] and in the perioperative period before liver transplantation [11]. In terms of recommended antibiotics for prophylactic treatment, norfloxacin [10, 13, 17, 21, 22] and ceftriaxone [13, 18, 21, 23] were mostly often recommended in the CPGs. Meanwhile, levofloxacin, ciprofloxacin, moxifloxacin, and trimethoprim–sulfamethoxazole (SXT) and rifaximin were also recommended.

**Table 3:**
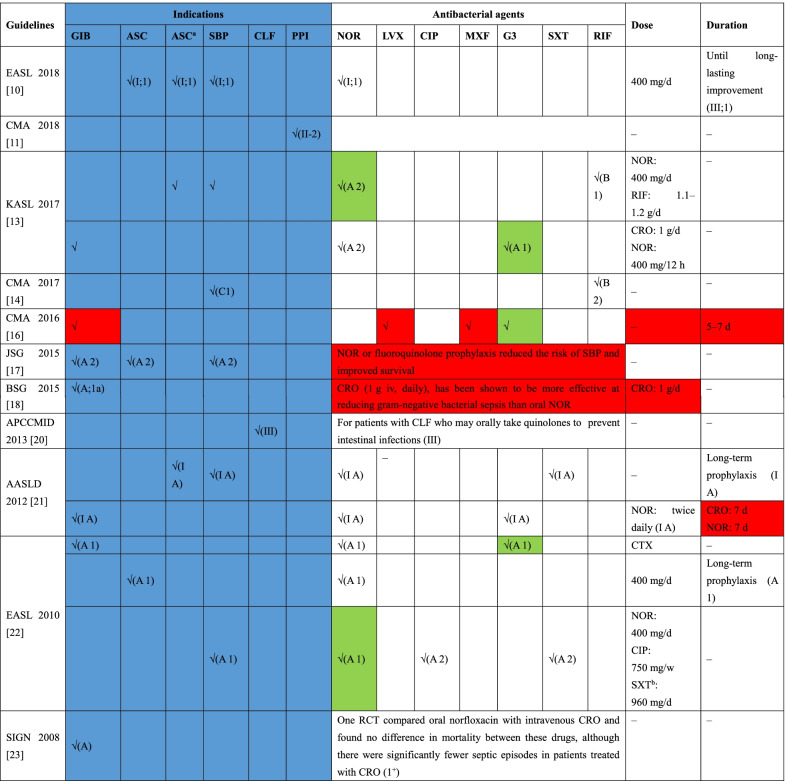
Indications and recommendations of prophylactic use of antibacterial agents for patients with liver cirrhosis or liver failure

Colour coding—blue: indications and recommendations of prophylactic use of antibacterial agents; red: recommendation provided without evidence level and strength; green: first-line prophylactic treatment

*CPGs* clinical practice guidelines, *EASL* European Association for the Study of the Liver, *CMA* Chinese Medical Association, *KASL* the Korean Association for the Study of the Liver, *JSG* Japanese Society of Gastroenterology, *BSG* the British Society of Gastroenterology, *APCCMID* Asia-Pacific Congress of Clinical Microbiology and Infection Consensus, *AASLD* the American Association for the Study of Liver Diseases, *SIGN* Scottish Intercollegiate Guidelines Network, *NOR* norfloxacin, *LVX* levofloxacin, *CIP* ciprofloxacin, *MXF* moxifloxacin, *G3* the third generation cephalosporins, *SXT* trimethoprim–sulfamethoxazole, *RIF* rifaximin, *CRO* ceftriaxone, *CTX* cefotaxime, *GIB* gastrointestinal bleeding, *ASC* ascites (the corresponding indication is patients with ascitic fluid protein lower than 15 g/L), *SBP* spontaneous bacterial peritonitis (the corresponding indication is patients recovered from spontaneous bacterial peritonitis), *CLF* chronic liver failure (the corresponding indication is patients with chronic liver failure), *PPI* perioperative prevention of infections (the corresponding indication is patients in perioperative period before transplantation)

^a^The corresponding indication is patients with ascitic fluid protein lower than 15 g/L + severe liver dysfunction or renal insufficiency or hyponatremia severe liver dysfunction or renal insufficiency/hyponatremia

^b^Co-trimoxazole (800 mg sulfamethoxazole and 160 mg trimethoprim daily, orally)

#### Prophylaxis of SBP

Six of the 14 CPGs [10, 13, 14, 17, 21, 22] reported recommendations for prophylaxis for SBP. Notably, five of 6 CPGs consistently recommended the use of norfloxacin (400 mg/d), except one [14] CPG from China. Furthermore, three CPGs [10, 21, 22] described the specific duration of norfloxacin treatment. Three CPGs recommended that patients who recovered from SBP should receive long-term norfloxacin prophylaxis until long-lasting improvement was observed. Additionally, ciprofloxacin [22], SXT [21] and rifaximin [13, 16] were also recommended for prophylaxis of SBP.

#### Prophylaxis in patients with low ascites protein levels and gastrointestinal bleeding

The regimens for prophylaxis in patients with low ascites protein levels were mentioned in 5 CPGs [10, 13, 17, 21, 22], which are generally consistent with the recommendations for SBP. However, ciprofloxacin and SXT were not recommended for patients with low ascites protein levels, although they were recommended for SBP in EASL 2010. Three CPGs recommended long-term antibiotic prophylaxis until long-term improvement [10] or disappearance of ascites [21, 22] in patients with low ascites protein levels. With regard to the recommendations for patients with gastrointestinal bleeding, 7 CPGs [13, 16–18, 21–23] consistently recommended the third-generation cephalosporins (G3) as prophylactic treatment, including two CPGs that recommended ceftriaxone 1 g/d for 7 days.

#### Which prophylactic strategy recommendations should clinicians follow?

For patients who have recovered from SBP or those with low total ascites protein levels, long-term prophylactic use of norfloxacin (400 mg/d) and ceftriaxone (1 g/day for 1 week) is generally recommended in the appraised CPGs based on high-quality evidence. In addition, cefotaxime is also recommended by EASL 2010 for the good penetration because of cefotaxime into ascites [24, 25]. Ciprofloxacin (750 mg once weekly, orally) and SXT (800 mg sulfamethoxazole and 600 mg trimethoprim daily, orally) are alternative antibiotics that are weakly recommended in EASL 2010. However, SXT and norfloxacin are both strongly recommended based on cost-effectiveness in AASLD 2012. Furthermore, rifaximin was also recommended in CMA 2017 and KASL 2017.

### Empirical antibiotic therapy for SBP and infections other than SBP

Recommendations for empirical antibiotic treatment were mentioned in 9 of 14 CPGs. Eight CPGs [10, 12–14, 19–22] suggested starting empirical antibiotics immediately when an infection is suspected or diagnosed. In addition, the environment (nosocomial *vs*. community acquired), bacterial resistance profiles and severity of infection should be taken into consideration to guide empirical antibiotics strategies. Among the 9 CPGs, 5 CPGs [10, 13, 14, 21, 22] detailed alternative drugs (Table 4). Five CPGs recommended G3 as the first-line choice for community-acquired SBP (CA-SBP), and 3 of them specifically recommended cefotaxime or ceftriaxone. However, EASL 2018 emphasized that G3 should be used in countries with low rates of bacterial resistance, while piperacillin/tazobactam (PTZ) or carbapenems (CARs) should be considered in countries with high rates of bacterial resistance. β-Lactam/β-lactamase inhibitor combinations (BLBLIs) should be chosen according to bacterial resistance profiles and the severity of bacterial infections, as recommended by EASL 2010, EASL 2018 and CMA 2017. In addition, CARs were only recommended by EASL 2018 and CMA 2017. Moderate-quality evidence supports the use of fluoroquinolones in CA-SBP for patients in the absence of recent fluoroquinolone antibiotic exposure [14, 21, 22]. Only CMA 2017 emphasized that empirical anti-gram-negative bacterial antibiotics should be combined with metronidazole to cover both gram-negative bacteria (GNB) and anaerobes. Moreover, only EASL 2018 emphasized that anti-gram-positive bacterial drugs, such as glycopeptides, daptomycin or linezolid, should be concluded in regimens for patients with healthcare-associated spontaneous peritonitis (HCA-SBP) and nosocomial spontaneous peritonitis (N-SBP) in areas with a high prevalence of gram-positive bacteria (GPB) infections.

**Table 4:**
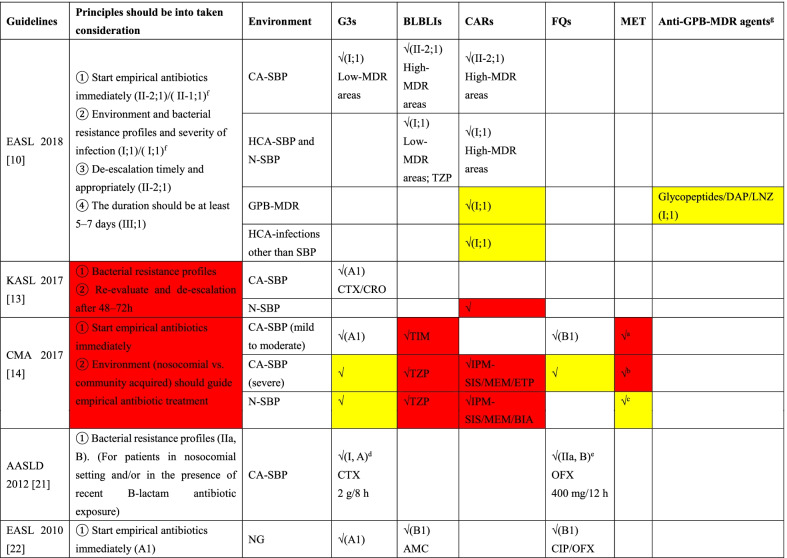
Principles and recommendations of empirical use of antibacterial agents for SBP and infections other than SBP in patients with liver cirrhosis or liver failure

Colour coding—yellow: combination regimen; red: recommendation provided without evidence level and strength

*SBP* spontaneous bacterial infections, *CPGs* clinical practice guidelines, *EASL* European Association for the Study of the Liver, *KASL* the Korean Association for the Study of the Liver, *CMA* Chinese Medical Association, *AASLD* the American Association for the Study of Liver Diseases, *G3s* the third generation cephalosporins, *BLBLIs* β-lactam/β-lactamase inhibitor combinations, *CARs* carbapenems, *FQs* fluoroquinolones, *MET* metronidazole, *GPB-MDR* gram positive bacteria multidrug-resistant, *CA-SBP* community-acquired spontaneous peritonitis, *HCA-SBP* healthcare-associated spontaneous peritonitis, *N-SBP* nosocomial spontaneous peritonitis, *HCA* healthcare associated, *DAP* daptomycin, *LNZ* linezolid, *CTX* cefotaxime, *CRO* ceftriaxone, *TIM* ticarcillin–clavulanic acid, *TZP* piperacillin–tazobactam, *IPM-SIS* imipenem–cilastatin, *MEM* meropenem, *ETP* ertapenem, *BIA* biapenem, *OFX* ofloxacin, *AMC* amoxicillin–clavulanic acid, *CIP* ciprofloxacin, *MDR* multidrug-resistant, *NG* not given

^a^MET should be combined with cefazolin/cefuroxime/cefotaxime/ceftriaxone/fluoroquinolones

^b^MET should be combined with ceftazidime/cefepime/fluoroquinolones

^c^MET should be combined with ceftazidime/cefepime, or tigecycline and colistin/polymyxin in some cases

^d^In the absence of recent β-lactam antibiotic exposure

^e^In the absence of recent fluoroquinolones antibiotic exposure

^f^Recommendations of infections other than SBP

^g^Carbapenem should be combined with glycopeptides or daptomycin or linezolid in areas with high prevalence of gram positive MDR bacteria

#### Which empirical strategy recommendations should clinicians follow?

In terms of empirical treatment, the recommended timing of initial therapy was consistent across 5 CPGs, which recommended starting empirical antibiotics immediately when a diagnosis of suspected SBP was made. As the frontline antibiotics used for empirical coverage of GNB in SBP, G3 was still recommended in recent CPGs for treating CA-SBP patients without recent G3 antibiotic exposure, despite the reported bacterial resistance to G3 [26] and unsatisfactory efficacy of CA-SBP [27]. BLBLIs and CARs should be chosen when taking bacterial resistance profiles and the severity of bacterial infections into consideration, as recommended by CPGs in the last 10 years [10, 13, 14, 22].

## Discussion

This is the first systematic appraisal of the methodological quality and recommendation assessment of published CPGs focused on antibacterial therapy strategies in patients with cirrhosis or liver failure using the validated AGREE II instrument. There was substantial variation in the quality of CPG development, and the overall quality was generally poor (median overall score, 56.3%). Considerable heterogeneity existed in the choice of treatment regimens for prophylactic or empirical treatment recommended by 14 CPGs. However, the principles and timing of the empirical treatment described in the 14 CPGs remained consistent.

### CPGs with a high overall score and CPGs scored highly in domain 4 should be followed by clinicians

Of the 14 CPGs, only three CPGs (KASL 2017, NICE 2016 and SIGN 2008) with an overall score above 80% were considered applicable without modification. In contrast, three CPGs from China (CMA 2016, CMA 2017 and CMA 2018) and two from the Asia-Pacific region (APASL consensus 2014 and APCCMID 2013) with an overall score less than 50% were considered inadequate for use in their current state. Furthermore, five CPGs (EASL 2018, EASL 2017, JSG 2015, BSG 2015, and AASLD 2012) scoring from 50 to 80% were recommended for use with modifications. In particular, one CPG (EASL 2010) from Europe that scored less than 50% was recommended for use with modifications because a higher score (92%) was obtained on domain 4, which is most frequently considered by healthcare practitioners to guide clinical practice. Similarly, 6 CPGs (EASL 2018, EASL 2017, KASL 2017, NICE 2016, AASLD 2012, and EASL 2010) that scored above 90% in domain 4 should also be given more attention by healthcare practitioners. Notably, the recommended SIGN 2008 scored 50% in domain 4 because the CPG mainly focuses on the assessment and management of variceal, nonvariceal, and colonic bleeding in adults [23]. Liver cirrhosis or liver failure patients with variceal bleeding account for a small proportion of the population to whom the guideline is meant to apply. Consequently, the antibiotic regimen recommendations for variceal bleeding in cirrhotic patients or liver failure patients in this study are nonspecific and ambiguous, which reasonably leads to relatively low domain 4 score.

### Shortcomings in some domains of included CPGs

Our systematic appraisal identified that there was a lack of attention to guideline applicability (domain 5, Applicability). Similarly, the involvement of patients or consumers in the development process (domain 2, Stakeholder Involvement) was often not fully addressed. It's worth noting that the scores of domain 5 and domain 2 are consistently low in other systematic reviews of CPGs on a wide range of healthcare topics [28–30]. Rigour of Development (domain 3) scored low in the present study, mainly due to poorly described methods for formulating recommendations in the development of CPGs. There was considerable variability (from 7.8 to 70.8%) across different CPGs concerning the domain of Editorial Independence (domain 6). The methods employed to avoid conflicts of interest are rarely described in most CPG content. Therefore, we strongly encourage guideline developers to consider the AGREE II framework when planning, developing and publishing CPGs.

### Prophylactic strategy based on literature evidence

For patients who have recovered from SBP or those with low total ascites protein levels, norfloxacin (400 mg/d), ceftriaxone (1 g/day for 1 week) and cefotaxime are generally recommended in the appraised CPGs based on high quality of evidence. According to two recently published RCTs, once-weekly ciprofloxacin was as effective as daily norfloxacin for the prevention of SBP in cirrhotic patients with ascites [31], and norfloxacin appears to increase survival rate of advanced cirrhotic patients with low ascites protein [32] while ciprofloxacin shows a unclear survival benefit [33]. At the same time, considering the increasing number of isolates that are resistant to fluoroquinolones and the risk of side effects of fluoroquinolones, Lombardi et al. concluded that SXT can be considered a safe and effective alternative, which has been affirmed by AASLD [34]. In terms of rifaximin, a meta-analysis demonstrated its fair efficacy [35], and an RCT reported that alternating norfloxacin and rifaximin showed superior prophylactic efficacy compared with monotherapy of norfloxacin [36]. Recently, EASL and other studies doubted the noninferiority of rifaximin compared to systemic antibiotics for SBP prophylaxis and advocated larger and well-conducted RCTs evidence to certify the efficacy of rifaximin [33, 34, 37]. We believe that the role of norfloxacin and G3 (ceftriaxone and cefotaxime) in the prophylaxis of SBP or ascites infections in patients with liver cirrhosis or liver failure is still unshakable in the face of the current evidence.

### Prophylactic strategy in the presence of MDR infections

Although CPGs and RCTs have demonstrated the cornerstone role of norfloxacin and G3 in prophylactic strategies, we also argue that the choice of prophylactic treatment strategies should be tailored according to the local epidemiology of bacterial resistance and access to medicine, as well as cost-effectiveness. Therefore, in patients colonized with fluoroquinolone-resistant GNB, alternative strategies such as rifaximin and SXT might be warranted to prevent SBP in areas with a high prevalence of fluoroquinolone-resistant bacteria. Several studies on cirrhosis concluded that fluoroquinolone prophylaxis was one of the main drivers of the spread of MDR infections [38–40]. However, according to a worldwide epidemiology study [7] focused on bacterial infections in patients with cirrhosis, SBP prophylaxis with quinolones was not a predictor of MDR bacterial infections. Therefore, until nonantibiotic options for SBP prophylaxis are available, what we can learn from this finding is that patients with an indication for primary or secondary SBP prophylaxis should be treated with quinolones. This strategy is also consistent with the latest CPG recommendation included in this study (EASL 2018). We encourage more high-quality clinical studies to provide evidence on prophylactic strategies in the presence of MDR infections.

### Empirical strategies based on literature evidence

G3, BLBLIs and CARs are widely recommended across CPGs. However, there is considerable uncertainty about which antibiotic therapy is better in patients with SBP according to a recent meta-analysis [41]. A systematic review demonstrated that a remarkable proportion of N-SBP is caused by MDROs [42]. Moreover, a high prevalence of GPB-MDROs was reported by intuitional centers in Europe [27] and Asia [43]. These findings suggested a need to cover the spectrum of GPB-MDROs in empirical treatment for N-SBP in areas with a high prevalence of GPB-MDROs, and carbapenem combined with glycopeptides, daptomycin or linezolid could be an appropriate treatment. A recent RCT [27] and a retrospective cohort study [44] also highlighted the importance of carbapenem combined with daptomycin and linezolid in the empirical antibiotic treatment of N-SBP.

For the management of infections other than SBP, only EASL 2018 strongly recommended carbapenems alone or in combination with other antibiotics in healthcare-associated infections other than SBP if high bacterial resistance to antibiotics were detected in the context. Most CPGs failed to provide adequate information on this issue due to a lack of high-level evidence.

### Future perspectives on diagnostics, biomarkers and antibiotic protocols for MDR bacterial infections

According to a worldwide epidemiology study, the global prevalence of MDR bacteria was as high as 34% in patients with cirrhosis [7]. Infections caused by MDR bacteria are associated with a higher risk of mortality than infections caused by susceptible bacteria, and are closely related to failure of antibiotic therapy and deterioration of liver function [39, 45]. Early identification and empirical treatment of MDR bacterial infections are important for reducing mortality. Therefore, suggestions on diagnostics, biomarkers and antibiotic protocols for MDR bacterial infections are important in the future, while the CPGs included in this study rarely mentioned this topic. Some suggestions for clinical practice could be drawn as follows:

#### New diagnostic approaches for MDR bacterial infections

In the future, the widespread use of novel rapid molecular diagnostic approaches focusing on pathogen detection and antimicrobial susceptibility tests should be implemented [46]. Some examples are matrix-assisted laser desorption ionization–time of flight (MALDI-TOF) MS, VitekMS, microarrays for detection of ESBLs and carbapenemases, whole-genome sequencing and next-generation sequencing (NGS) technologies. These diagnostic approaches can contribute to choosing the best timing of appropriate antibiotic treatment strategy and/or de-escalation, and shorten the overall duration of antibiotic treatment [47].

#### Biomarker for MDR bacterial infection

In recent years, most host-derived tests for bacterial infections focused on peptide-based biomarkers have been applied to clinical practice, such as C-reactive protein (CRP), procalcitonin (PCT), tumour necrosis factor-related apoptosis-inducing ligand (TRAIL), protein-10 (IP-10) and a combination of these biomarkers [48–50]. Additionally, with the emergence of molecular diagnostic techniques, promising lipid biomarker such as LPS, LTA, LBP and the immunologic biomarker soluble CD14 subtype, known as presepsin, for the detection of bacterial infections have been found [51, 52]. Identifying specific and dynamic biomarkers of MDR bacterial infection that can be used for follow-up and for determining outcome will help treatment success. However, there is still a need to conduct research to find biomarkers of MDR bacterial infection in patients with cirrhosis or liver failure.

#### Antibiotic protocols for MDR bacterial infections

The use of antibiotics needs to be wise and judicious, since a ‘one-size-fits-all’ approach is not advisable. To minimize the increasing development of antibiotic resistance, a series of programmes should be taken into account: (1) antibiotic stewardship programmes should be enhanced to improve the rational use of antibiotics both in hospital and community settings [7]; (2) regular MDRO screening of patients with liver cirrhosis during antibiotic prophylaxis should be considered, especially in areas with high MDRO rates [53]; (3) antibiotic prophylaxis mainly with poorly absorbed antibiotics, such as selective intestinal decontamination, should be considered; and (4) given that antibiotic PK/PD is rarely studied and that drug exposure is unpredictable in patients with liver dysfunction, therapeutic drug monitoring, whenever possible, should be performed to design individualized dosage regimens.

### Strengths and limitations

This is the first systematic appraisal to identify and evaluate CPGs on bacterial infections in patients with cirrhosis or liver failure. The main strength of our systematic appraisal lies in the combination of methodological quality appraisal and content analysis of recommendations. A study limitation is that the AGREE II scoring system relies on the intelligibility and comprehensiveness of the CPGs’ reporting rather than reflects the methodological quality or strength of the evidence. Therefore, when we ranked CPGs based on overall scores evaluated by AGREE II, there was potential bias from misinterpretation derived from the formulation and wording of recommendations. However, we could pay more attention to the recommendations provided by CPGs that scored higher on AGREE II criteria, especially those with high scores on domain 4. Another limitation is in that only English- and Chinese-language CPGs were reviewed, and the search strategy may have failed to identify high-quality CPGs reported in other languages.

## Conclusions

The methodological quality of CPGs focused on patients with cirrhosis or liver failure evaluated by the AGREE II instrument is generally poor. Regarding to recommendations, fluoroquinolones (norfloxacin and ciprofloxacin), G3 (ceftriaxone and cefotaxime), and SXT are recommended for prophylactic treatment appropriately. G3, BLBLIs, and CARs are recommended as empirical treatment strategies according to the local epidemiology of bacterial resistance. We call for further improvement in the ‘Applicability’ domain in CPG development, and further researches are needed to strengthen the evidence-based recommendations to reach a consensus on the management of bacterial infections for patients with liver cirrhosis or liver failure.

## Supplementary Information

**Additional file 1: Appendix S1.** Search strategy.

**Additional file 2: Appendix S2.** Grading systems used in the appraised CPGs.

## Data Availability

Data and materials during the current study are available from the corresponding author on reasonable request.

## Abbreviations

SBP: Spontaneous bacterial peritonitis
MDROs: Multi-drug resistance organisms
CPG: Clinical practice guideline
CNKI: National Knowledge Infrastructure
NICE: the National Institute for Health and Care Excellence
GIN: Guidelines International Network
SIGN: Scottish Intercollegiate Guidelines Network
NHMRC: National Health and Medical Research Council
NZGG: New Zealand Guidelines Group
PRISMA: Preferred Reporting Items for Systematic Reviews and Meta-Analyses
AGREE: Appraisal of Guidelines for Research and Evaluation
GRADE: Grading of Recommendations Assessment, Development and Evaluation
SXT: Trimethoprim–sulfamethoxazole
G3: the Third-generation cephalosporins
PTZ: Piperacillin/tazobactam
CARs: Carbapenems
BLBLIs: β-Lactam/β-lactamase inhibitor combinations
GNB: Gram-negative bacteria
HCA-SBP: Healthcare-associated spontaneous peritonitis
N-SBP: Nosocomial spontaneous peritonitis
GPB: Gram-positive bacteria
EASL: European Association for the Study of the Liver
CMA: Chinese Medical Association
LFALG: Liver Failure and Artificial Liver Group
CSID: Chinese Society of Infectious Diseases
SLDALG: Severe Liver Disease and Artificial Liver Group
CSH: Chinese Society of Hepatology
KASL: The Korean Association for the Study of the Liver
CSG: Chinese Society of Gastroenterology
CSE: Chinese Society of Endoscopy
JSG: Japanese Society of Gastroenterology
BSG: The British Society of Gastroenterology
APASL: Asian Pacific Association for the Study of the Liver
APCCMID: Asia-Pacific Congress of Clinical Microbiology and Infection Consensus
AASLD: The American Association for the Study of Liver Diseases
SIGN: Scottish Intercollegiate Guidelines Network
ACC/AHA: The American College of Cardiology and the American Heart Association Practice Guidelines
