# Supplementary material for: Evaluation of the current guidelines for antibacterial therapy strategies in patients with cirrhosis or liver failure
Source: BMC Infect Dis. 2022 Jan 4;22:23. doi: 10.1186/s12879-021-07018-2 (PMC8725452; doi:10.1186/s12879-021-07018-2)
Supplement: Supplementary file 1 — Additional file 1: Appendix S1. Search strategy. [file 12879_2021_7018_MOESM1_ESM.docx]

**Search strategy**

#1 Liver Cirrhosis[Mesh Terms]

#2 liver Cirrhos* [Text Word]

#3 Hepatic Cirrhos*[Text Word]

#4 liver Fibros*[Text Word]

#5 Liver Failure[Mesh Terms]

#6Hepatic Failure[Text Word]

#7 End Stage Liver Disease[Mesh Terms]

#8 Chronic Liver Failure*[Text Word]

#9 or/#1-8

#10Bacterial Infections[Mesh Terms]

#11Bacterial infectio*[Text Word]

#12 Anti-Bacterial Agents[Mesh Terms] OR Antibacterial Agents[Text Word]

#13 Bacteriocid* [Text Word]

#14 Bacterial* [Text Word]

#15 Anti Mycobacterial Agents[Text Word]

#16Antibiotic*[Text Word]

#17 mycoses[MeSH Terms] OR mycoses[Text Word]

#18 fung*[Text Word]

#19 antifungal [Text Word]

#20 antifungal agents[MeSH Terms]

#21 or/#10-20

#22 Guideline[MeSH Terms]

#23 Guideline*[Text Word]

#24 or/#22-23

#25 #9 AND #21 AND #24
