# Supplementary material for: Evaluation of the current guidelines for antibacterial therapy strategies in patients with cirrhosis or liver failure
Source: BMC Infect Dis. 2022 Jan 4;22:23. doi: 10.1186/s12879-021-07018-2 (PMC8725452; doi:10.1186/s12879-021-07018-2)
Supplement: Supplementary file 2 — Additional file 2: Appendix S2. Grading systems used in the appraised CPGs. [file 12879_2021_7018_MOESM2_ESM.docx]

Appendix S2 Grading systems used in the appraised CPGs

| CPGs | Grading system applied | Codes of Evidence and Recommendation | |
| --- | --- | --- | --- |
|  |  | Level | Strength |
| EASL 2018 | GRADE | I, II-1, II-2, II-3, III | Strong(1), Weak(2) |
| CMA 2018 | GRADE | I, II-1, II-2, II-3, III | NR |
| EASL 2017 | GRADE | I, II-1, II-2, II-3, III | Strong(1), Weak(2) |
| KASL 2017 | GRADE | A, B, C | Strong(1), Weak(2) |
| CMA 2017 | GRADE | A, B, C | Strong(1), Weak(2) |
| NICE 2016 | GRADE | I, II-1, II-2, II-3, III | Strong(1), Weak(2) |
| CMA 2016 | GRADE | A, B, C | Strong(1), Weak(2) |
| JSG 2015 | GRADE | I, II-1, II-2, II-3, III | Strong(1), Weak(2) |
| BSG 2015 | Oxford | 1a, 1b, 1c, 2a, 2b, 2c, 3a, 3b, 4, 5 | A, B, C, D |
| APASL consensus 2014 | Oxford | 1a, 1b, 1c, 2a, 2b, 2c, 3a, 3b, 4, 5 | A, B, C, D |
| APCCMID 2013 | GRADE | I, II-1, II-2, II-3, III | NR |
| AASLD 2012 | ACC/AHA | A, B, C | I, II, IIa, IIb, III |
| EASL 2010 | GRADE | A, B, C | Strong(1), Weak(2) |
| SIGN 2008 | SIGN | 1^++^, 1^+^, 1^-^, 2^++^, 2^+^, 2^-^ 3, 4 | A, B, C, D |

CPGs: clinical practice guidelines; EASL: European Association for the Study of the Liver; CMA: Chinese Medical Association; KASL: the Korean Association for the Study of the Liver; NICE: National Institute for Health and Care Excellence; JSG: Japanese Society of Gastroenterology; BSG: the British Society of Gastroenterology; APASL: Asian Pacific Association for the Study of the Liver; APCCMID: Asia-Pacifc Congress of Clinical Microbiology and Infection Consensus; AASLD: the American Association for the Study of Liver Diseases; SIGN: Scottish Intercollegiate Guidelines Network. GRADE: Grading of Recommendations Assessment, Development and Evaluation; ACC/AHA: the American College of Cardiology and the American Heart Association Practice Guidelines. NR: Not report.
